# Multiplexed CRISPR Assay for Amplification-Free Detection of miRNAs

**DOI:** 10.3390/bios15060346

**Published:** 2025-05-29

**Authors:** P. I. Thilini De Silva, Keshani Hiniduma, Rachelle Canete, Ketki S. Bhalerao, Sherif M. Shawky, Hansana Gunathilaka, Jessica L. Rouge, Islam M. Mosa, David C. Steffens, Kevin Manning, Breno S. Diniz, James F. Rusling

**Affiliations:** 1 Department of Chemistry, University of Connecticut, Storrs, CT 06269, USAketki.bhalerao@uconn.edu (K.S.B.); sherif_mohamed.shawky_abdou@uconn.edu (S.M.S.); jessica.rouge@uconn.edu (J.L.R.); islam.mosa@uconn.edu (I.M.M.); 2 Department of Psychiatry, UConn Health, Farmington, CT 06030, USA; steffens@uchc.edu (D.C.S.); diniz@uchc.edu (B.S.D.); 3 UConn Center on Aging, UConn Health, Farmington, CT 06030, USA; 4 School of Chemistry, National University of Ireland at Galway, H91 TK33 Galway, Ireland; 5 Department of Surgery and Neag Cancer Center, UConn Health, Farmington, CT 06030, USA; 6 Institute of Material Science, University of Connecticut, Storrs, CT 06269, USA

**Keywords:** CRISPR, Alzheimer biomarkers, miRNAs, multiplexed assay, fluorescence

## Abstract

CRISPR-Cas proteins from bacteria are powerful tools for gene editing and molecular diagnostics. Expanding capacity of CRISPR to low cost, multiplexed assays of biomarkers is a key to future disease diagnostics, since multiple biomarker detection is essential for reliable diagnostics. Herein we describe a multiplexed assay in a 3D-printed 96-well plate with CRISPR-Cas13a immobilized in each well to target three circulating blood biomarker microRNAs (miRNAs 34c-5p, 200c-3p, and 30e-5p) for Alzheimer’s disease (ALZ). Immobilized Cas13a is equipped with different crRNAs complementary to each miRNA target. MiRNA binding to crRNA complements activates the collateral RNase activity of Cas13a, cleaving a quenched fluorescent reporter (RNaseAlert) with fluorophore and quencher connected by an RNA oligonucleotide to enable fluorescence measurements. We achieved ultralow limits of detection (LOD) of 0.74 fg/mL for miRNA 34c-5p, 0.70 fg/mL for miRNA 30e-5p, and 7.4 fg/mL for miRNA 200c-3p, with dynamic ranges from LODs up to about 1800 pg/mL. The accuracy of the assay was validated by spike-recovery studies and good correlation of levels of patient plasma samples vs. a referee method. This new approach provides selective, sensitive multiplex miRNA biosensing, and simultaneously accommodates analysis of standards and controls.

## 1. Introduction

Molecular biomarkers, including proteins and nucleic acids [1,2], play a vital role in disease diagnostics and monitoring patient health and therapeutic outcomes. Accurate analyses of multiple biomarkers, as opposed to single biomarkers, offer objective measurements of physiological and pathological processes. However, biomarker assays are currently underutilized due to the unavailability of assays in clinics and hospitals, partly due to the cost and complexity of commercial options [3]. Disease detection utilizing CT, MRI, and PET imaging can be used, but high cost and low sensitivity for early detection limit their effectiveness, e.g., for cancers and Alzheimer’s disease [4,5,6].

Nevertheless, huge advances in molecular diagnostic test research have been made over the past 10–15 years, propelled by breakthroughs in microfluidics, biotechnology, and molecular biology [7,8,9]. For example, simple, low-cost microfluidic arrays [10,11,12] can achieve limits of detections below 1 fg/mL for multiple proteins in fast, semi-automated immunoassays [13,14]. New methodologies involving CRISPR-based assays [15], surface plasmon resonance (SPR) [16,17], and methylation signatures of blood-circulating cancer cells [18] also provide high sensitivity and specificity for biomarker detection. Such developments provide the power of early clinical detection of illnesses that can lead to faster and more effective, less invasive treatments [19]. Cutting-edge technologies facilitated by low-cost point-of-care procedures in a hospital, clinic, or physician’s office have the potential to transform disease management and enhance positive patient outcomes by enabling early detection [20,21].

CRISPR-Cas was first identified as a bacterial defense mechanism against viruses. CRISPR (Clustered Regularly Interspaced Short Palindromic Repeats) and Cas (CRISPR-associated) proteins work together to recognize and cleave specific nucleic acid regions [22]. CRISPR-Cas systems were identified and developed in the early 2010s, and have found wide use in science, agriculture, and medicine [21,23]. In diagnostic tests, CRISPR offers simpler and more direct analyses than PCR for nucleic acids, and has better sensitivity [24,25].

Bioanalytical CRISPR methods such as SHERLOCK (Specific High-sensitivity Enzymatic Reporter unLOCKing) and DETECTR (DNA Endonuclease Targeted CRISPR Trans Reporter) have been developed to accurately identify bacterial and viral infections [26], dengue virus [27], zika virus [28], and SARS-CoV-2 [29,30,31]. However, current drawbacks include the requirement for amplification of analytes to achieve the necessary sensitivity, and the lack of a general approach to multiple analyte detection, or multiplexing.

There is a large diagnostic advantage to measuring multiple biomarkers in a single experiment [20,32]. For example, the measurement of four prostate-cancer biomarkers in patient serum provided greatly improved diagnostic performance in predicting whether a patient needs a biopsy compared to the clinically used single protein prostate specific antigen [13]. Multiplex assays can also detect co-infections and track multiple pathogens in infectious diseases [33,34,35]. For example, a multiplexed CRISPR assay can detect a panel of 21 respiratory viruses, including SARS-CoV-2 variants, thereby providing a general test for virus identification [36]. Multiplexed CRISPR technology has also been used to integrate comprehensive disease profiles in a single test [37,38,39].

**Figure 1:**
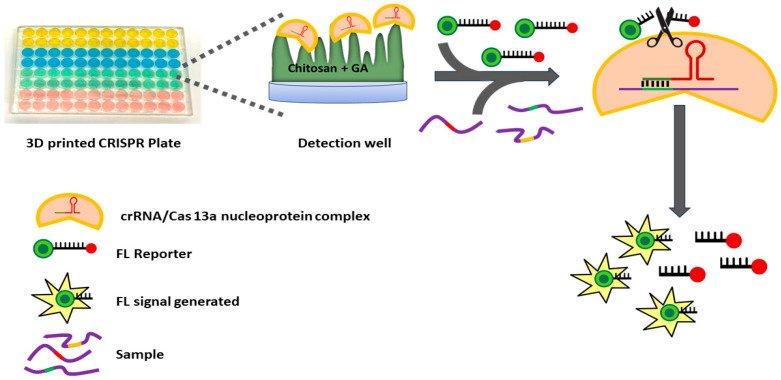
Schematic showing steps for the CRISPR assay in the 3D-printed well plate.

Herein we describe a simple mix-and-read, high-sensitivity, high-specificity, CRISPR/Cas miRNA assay system that features a general approach to multiplexing and is illustrated by the detection of a miRNA panel for Alzheimer’s disease (ALZ) detection (Figure 1). The platform is a 3D-printed 96-microtiter well plate in which Cas31a protein and different CRISPR RNAs (crRNA/Cas13a nucleoprotein complex) complementary to target miRNAs are chemically immobilized onto a hydrogel in specific wells in a generally applicable approach to multiplexing. These immobilized CRISPR plates are storable for at least one week. For the assay, samples or standards containing miRNAs mixed with fluorescent reporter are then added to each well to produce individual signals for each target. We illustrate assay performance by detecting three plasma miRNAs (miR-34c-5p, miR-30e-5p, and miR-200c-3p) previously linked to early-stage ALZ [40,41,42]. When the CRISPR-cas13a’s bind their specific targets in individual wells, the resulting non-discriminate cas13a RNAse activity cleaves poly-RNA links in the reporter between fluorophore and quencher to produce fluorescence in each well measured by a plate reader. The RNA reporter is a short, single-stranded RNA molecule labeled with a fluorescent dye at one end and a quencher at the other. In its intact state, the close proximity of the fluorophore and quencher enables Fluorescence Resonance Energy Transfer (FRET), effectively suppressing fluorescence emission. Upon the specific binding of the CRISPR RNA (crRNA) to its complementary miRNA target, Cas13a is activated and exhibits collateral RNase activity, randomly cleaving RNA molecules in the solution, including the reporter. This cleavage separates the fluorophore from the quencher, restoring fluorescence that can be detected upon excitation at the dye’s characteristic wavelength. The resulting fluorescence intensity is directly proportional to the concentration of the target miRNA, providing a quantitative, real-time readout of its presence. Calibration standards can be measured in the same plate at the same time as samples. This approach provides highly sensitive and selective assays for multiple miRNAs in plasma and other fluids without amplification.

## 2. Materials and Methods

All reagents and chemicals were analytical grade. Chitosan (MW 448869), and glutaraldehyde, glycerol, and dithiothreitol solution (DTT) 1M were from Sigma Aldrich (St. Louis, MO, USA). RNaseAlert^TM^ Lab Test Kit v2, SUPERase•In™ RNase Inhibitor (20 U/μL) UltraPure™ 1 M Tris-HCI Buffer pH 7.5, UltraPure^TM^ water, was from Thermo Fisher (Waltham, MA, USA). Cas13a 1mg/mL (CAS 13a-200) was from MCLAB. miR-34c-5p, miR-200c-3p, miR-30e-5p, crRNA-miR-34c-5p, crRNA-miR-200c-3p, crRNA-miR-30e-5p were from Integrated DNA Technologies (Coralville, IA, USA). Pooled human plasma was from Innovative™ Research (Novi, MI, USA). The storage buffer was Tris-HCl (pH 7.5, 1 M), NaCl (5 M), glycerol, MgCl_2_ (0.25 M), and DTT (1 M), in ultrapure water. Fluorescence was measured using a BioTek^®^ Multi-Mode Microplate Reader (Agilent, Santa Clara, CA, USA) and Gen5^TM^ software, version 2.0. A Form Labs Form 3B+ 3D printer and Clear Resin (RS-F2-GPCL-04) were used for 3D printing. Alzheimer’s patient plasma samples were from UCONN Health Center (Farmington, CT, USA), (IRB approval 19-214S-1).

Well plates were printed using predetermined optimal 50 mL volume on a FormLabs Form 3B stereolithographic 3D printer (FormLabs, Somerville, MA, USA). The polyacrylate polymer used strongly chemisorbs chitosan [20] (see below) and was critical to assay sensitivity. Printed arrays were cleaned by sonicating and washing in isopropanol for 10–15 min to remove uncured resin, followed by air drying and curing at 60 °C for 30 min.

**Figure 2:**
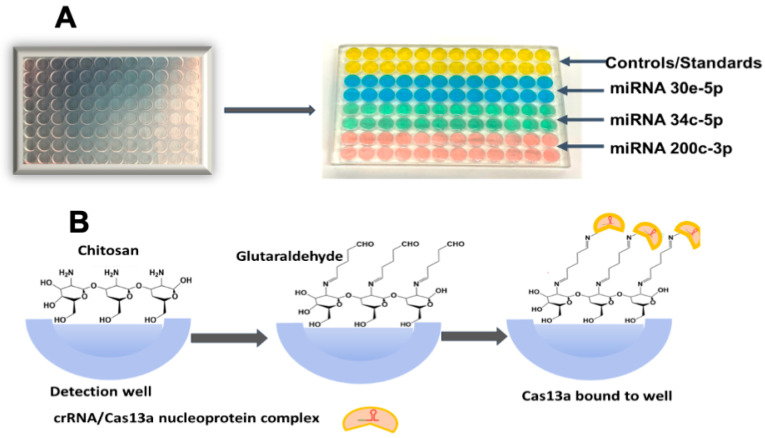
Three-dimensional-printed polyacrylate plates for multiplexed CRISPR miRNA assay: (**A**) 96-well microfluidic CRISPR plate with 50 uL vol. microwells (on left), and analytical CRISPR plate after immobilizing the desired crRNA/Cas13a’s in the wells. (**B**) Binding of Cas13 into wells by chemical attachment to chemisorbed chitosan hydrogel.

As shown in Figure 2, to each microwell 50 µL 0.5 mg/mL chitosan in 0.05 M HCl was added and incubated at room temperature for 3 h. Solution was removed by tapping the plate upside down, then it was left to dry overnight in a vacuum to provide hydrogel coats in each microwell. Then, wells were filled with 3% glutaraldehyde and incubated 1 h. to form Schiff’s bases between amines on chitosan and glutaraldehyde [43]. Excess solution was drained by inverting the plate and tapping, followed by vacuum drying 1 h. Separate microwells were then reacted with crRNA/Cas13a nucleoprotein complex of miRNAs 30e-5p, miRNA34c-5p, and miRNA 200c-3p. Cas13a/crRNA/nucleoprotein complex mixtures were prepared by mixing 400 mM Tris-HCl buffer (5 µL), ultrapure water (35 µL), Cas13a (63.3 µg/mL) (5 µL), SUPERase•In RNase Inhibitor (2.5 µL), and the appropriate CRISPR RNA (crRNA) (2.5 µg/mL) (2.5 µL), and incubating for 12 min. at 37 °C. The required Cas 13a/crRNA (50 µL) were then added to their respective wells and incubated overnight at 4 °C. This process facilitates a reaction between free glutaraldehyde and amines on the Cas13a protein in the mixture with the chitosan hydrogel already chemisorbed in the wells. The quantification of immobilized Cas13a in the microwells was carried out using the Bicinchoninic Acid (BCA) total protein assay [44], revealing 37 × 10^11^ Cas13a molecules/well. Excess crRNA/Cas13a was removed by tap drying, and washed with pH 7.5 (400 mM) Tris-HCl buffer. The reaction mixture contained 400 mM Tris-HCl (5 µL), RNase-free H_2_O (38.25 µL), SUPERase•In RNase Inhibitor (1.25 µL), RNaseAlert reporter (2 µM) (2.5 µL), pooled human plasma (0.5 µL), and the respective target miRNA sample (2.5 µL) (total 50 μL per well based on the optimum volume for best assay performance, as shown in Figure S1), were kept in microcentrifuge tubes in an ice bath while preparing to add them to wells.

**Figure 3:**
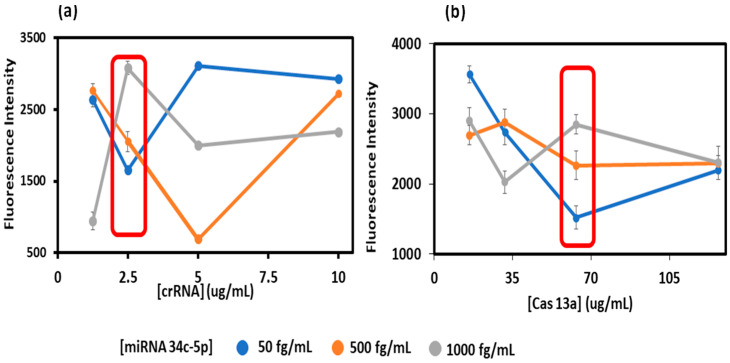
Optimization plots for (**a**) crRNA and (**b**) Cas13a with respect to the miRNA 34c-5p biomarker. The red boxes indicates the optimal concentrations, where the signal increase per unit concentration is the largest.

In the assay procedure, optimized amounts of Cas13a and crRNA are added to 400 mM TRIS-HCl buffer, RNase-free water, Cas13a, inhibitor, and crRNA, and incubated at 37 °C for 12 min. This mixture was added to respective wells in the well-plate pre-functionalized with chitosan and glutaraldehyde. For crRNA optimization, the reaction mixture was prepared with different crRNA concentrations (1.25 to 10 μg/mL) with a ‘maximum Cas13a concentration’ (127 ug/mL) to ensure a maximum dynamic range of analyte concentrations was captured. Then, the assay was conducted across different analyte miRNA concentrations within the desired dynamic range [miRNA30e-5p] = (50, 500 and 1000 fg/mL). As mentioned above, the crRNA concentration that provided the largest signal separation in triplicate, and between low and high miRNA levels in the correct level of low to medium to high, was used as the optimized crRNA concentration (Figure 3a). Using this optimized crRNA concentration, the optimize dCas13a concentration was used as the largest signal separation between low and high miRNA (Figure 3b).

## 3. Results

### 3.1. Assay Optimization

The assay was designed to be conducted in 3D-printed 96-well plates with individual crRNA/Cas13a’s chemically bound to chemisorbed chitosan in each well. CrRNA optimization was carried out by testing concentrations of the miRNA in the range of 1–10 μg/mL with maximum Cas13a (127 μg/mL) to maximize signal separation, e.g., shown for miRNA30e-5p detection (Figure 3a) [20,41]. The best crRNA was selected for each of the analyte miRNAs, as the value that provided increased fluorescence from low to high values in the correct order. This crRNA level was then used to optimize Cas13a (range 16–127 μg/mL) using the same criteria (Figure 3b).

### 3.2. Calibration and Validation

**Figure 4:**
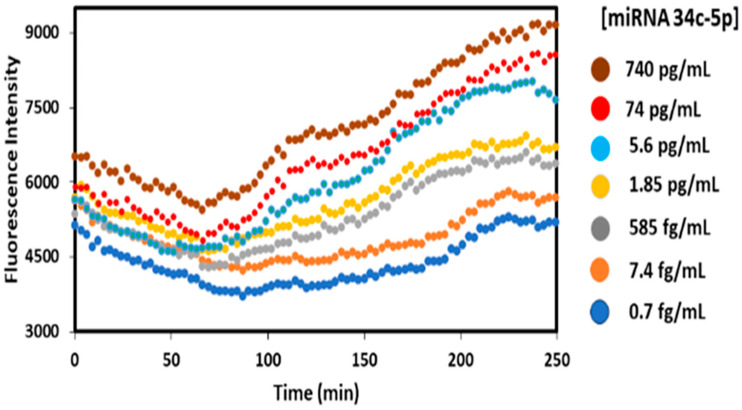
Fluorescence plate reader measurement using 490 and 520 nm as excitation and emission wavelengths for different concentrations of miRNA 34c-5p at 37 °C. For calibrations and sample analyses, emission intensity was measured after subtracting the plateau readings (90–100 min) from exponential phase points (140–150 min). All concentration-dependent signals were baseline-corrected by subtracting the corresponding control signal.

For each miRNA, calibrations were carried out using the optimized parameters described above over the dynamic range of the three miRNAs in solutions of 0.7 fg/mL to 1756 pg/mL miRNA, complementary to the immobilized crRNA/Cas-13a in the well, along with 2.5 uL of fluorescence reporter in 0.1% pooled human plasma for all samples. Figure 4 shows raw data from a typical calibration experiment showing fluorescent output from the plate reader using 490 and 520 nm as excitation and emission wavelengths, respectively, and nm incident light and measured fluorescence output at 520 nm vs. time after the start of the CRISPR Cas13a RNA cleavage activity upon the addition of an miRNA analyte. At the beginning of the experiment, fluorescence decays a bit, attributed to the addition of the miRNA reaction mixture that had been stored in an ice bath to stabilize the miRNAs. The small decrease is related to the time required for temperature equilibration in the well, and when the solution is equilibrated the reported cleavage rection that releases florescence increases until most of it is cleaved.

**Figure 5:**
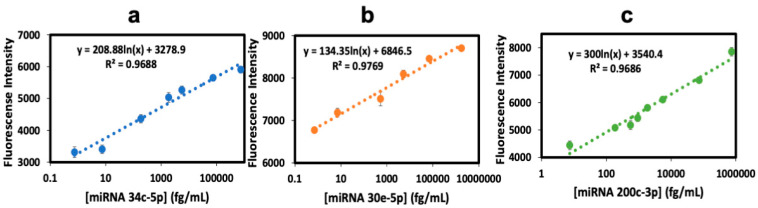
Calibration plots in 0.1% pooled human plasma using the fluorescence plate for simultaneous fluorescent detection of (**a**) miRNA 34c-5p, (**b**) miRNA 30e-5p, and (**c**) miRNA 200c-3p (error bars: SD for n = 3).

While the fluorescent traces are somewhat noisy, differences between the fluorescence rise at 140–150 min after subtracting the plateau at 90–100 min were quite reproducible and were used to prepare calibration curves. Differences between the 250 min and 90–100 min signals gave qualitatively similar calibrations, but added 100 min to the assay time. Fluorescence (FL) intensity differences at 140–150 minus 90–100 min as indicated were plotted against the concentration (Figure 5) to determine the linear dynamic range and the limit of detection (LOD), as 3× standard deviation (SD) of the blank above the blank signal for each miRNA.

Calibration graphs in Figure 5 show good linearity with the corresponding equations, and (r^2^) correlation coefficient values of 0.9688 for miRNA-34c-5p, 0.9769 for miRNA-30e-5p, and 0.9686 for miRNA-200c-3p. Calibration data show small standard deviations for each miRNA. Linear dynamic ranges are 0.74 fg/ mL–740 pg/mL (0.1 fM–100 pM) for miRNA 34c-5p, 0.7 fg/mL–1756 pg/mL (0.1 fM–250 pM) for miRNA 30e-5p, and 7.4 fg/mL–74.1 pg/mL (1 fM–10 pM) for miRNA 200c-3p, with the average relative standard deviation of ±5% plate to plate (n = 6). LODs obtained were 0.74 fg/mL for miRNA 34c-5p, 0.7 fg/mL for miRNA 30e-5p, and 7.4 fg/mL for miRNA 200c-3p. Reproducible signal intensity was found for the three microwells allotted to each miRNA within a single plate (Figure 5), giving an average standard deviation of ±3% well-to-well. Spike recovery tests (Table 1) gave values that were all within the acceptable analytical range of 100 ± 20% for biomedical assays [45]. A cross-reactivity assay involved testing one miRNA against the crRNA of the other two biomarkers and analyzing the resulting signals. Cross-reactivity was below 5% in all cases (Table 2, grey areas for no cross-reactivity possible).

Spike recovery studies were conducted by spiking pooled human plasma with known concentrations (70 fg/mL, 850 fg/mL, 0.35 pg/mL, and 35 pg/mL) of each analyte miRNA and measuring blank-subtracted fluorescence (FL) intensities. Calibrations in pooled human plasma were used to determine the concentration of each analyte. Spike recovery % was established by comparing the known spike concentrations with the concentration found from the assay. Cross-reactivity between different miRNAs in pooled human plasma was established by testing one miRNA against the crRNA of the remaining two markers under study.

Elevated levels of miRNAs 30e, 34c, and 200c have been reported in human blood and brain in ALZ patients, and which have been shown to be highly correlated with early and later stages of the disease [38,39,40]. Preliminary studies showed that it was necessary to extract the RNAs from plasma to remove interferences in the assay, so all results below involving patient samples are shown for RNA extracted from plasma.

Samples from eight Alzheimer’s patients were analyzed in a blind study. miRNAs were extracted using the mirVana™ PARIS™ RNA and Native Protein Purification Kit (Thermo Fisher), and the optimized fluorescence-based assay was performed in the 96-well plate to detect three miRNA analytes.

Of eight patient samples analyzed, six were diagnosed as AD-positive, while the remaining two negative samples exhibited very low concentrations of all three miRNAs (Table 3). To further validate the assay, results were compared with our developed ECL-based CRISPR assay for the eight patient samples displayed in the Supplementary Materials, Figure S2, showing slopes near 1.0 and intercepts close to zero for each miRNA, indicating excellent agreement between the two methods. Additionally, R^2^ values close to 1.0 further confirm an excellent correlation.

## 4. Discussion

The mix-and-read CRISPR assay described above features immobilized Cas13a protein loaded with corresponding crRNAs in a 96-well, 50 mL plate designed to detect multiple, unamplified miRNAs in plasma or serum samples. It is easy-to-use, low-cost (~USD 0.70 per printed plate and USD 2.80 for reagents per plate), specific, and highly sensitive using fluorescence. The new features for this CRISPR assay that is applicable to a collection of miRNAs are (1) the stable binding of Cas13a into the 50-mL wells of the 3D-printed polyacrylate 96-well plate, allowing the pre-storage of loaded plates for one week in a refrigerator, (2) a reaction mixture including the sample and fluorescent reporter that enables a one-step addition for the detection measurements, and (3) very high sensitivity that can directly address miRNAs in clinical plasma and serum samples without preamplification.

Excellent assay calibrations (Figure 4), spike recoveries (Table 1), and low cross reactivities (Table 2) were demonstrated for the detection of three known miRNA biomarkers for Alzheimer’s disease (ALZ) in plasma. This is important since blood miRNAs are being intensely investigated as likely biomarkers for AD, since about 70% of the currently known miRNAs are expressed in the brain and involved in neurological processes [46,47,48,49]. Our new multiplexed CRISPR assay can be readily applied to any set of RNA molecules. Our assay offers potential benefits and distinct advantages in sensitivity and multiplexity over established miRNA detection techniques, including genome sequencing and PCR methods [50,51]. While PCR tests are regarded as gold standards for RNA detection, they do not directly detect miRNA concentrations, and require specialized instrumentation as well as amplification of the targets for use in plasma and serum. Also, PCR applications at low-resource sites, such as third-world countries, is restricted due to costly equipment and the need for specialized expertise [51,52]. The earlier SHERLOCK method utilizing CRISPR requires amplified RNA or DNA by recombinase polymerase amplification (RPA). Assay time is about 1–2 h, and does not feature multiplexing [15,51]. miSHERLOCK and CARMEN are multiplexing CRISPR systems, which require comparably large amounts of sample and additional amplification steps [15,38,52].

In the new CRISPR assay procedure described above, crRNA/Cas13a is chemically bound in fully active form to chitosan hydrogel in microwells of a 96-well plate, representing to our knowledge the first immobilized CRISPR platform. Previous work [41] in our lab showed that chitosan is strongly chemisorbed onto the 3D-printed polyacrylate polymer used. The film contains 98% water, resulting in a highly porous hydrogel that provides a high surface area for binding the crRNA/Cas13a complex, and a porous, water-rich network that allows the efficient internal mass transportation of water soluble molecules in the wells. Well plates with immobilized cas13a can be refrigerated at 4 °C for up to five days and used when needed. However, it is crucial to eliminate or inactivate any RNases present that can cleave reporter molecules. In our method, we recommend using an RNAse inhibitor during sample preparation to deactivate the RNases that may be present.

The new CRISPR miRNA assay as presented here achieves extremely low limits of detection (LODs) for multiple analytes without RNA amplification. Detection limits were 0.74 fg/mL for miRNA 34c-5p, 0.7 fg/mL for miRNA 30e-5p, and 7.4 fg/mL for miRNA 200c-3p. The assay can be applied to many more miRNAs than in this first example. Due to this high sensitivity, plasma samples need to be diluted so that concentration levels of target miRNAs fall within the assay’s dynamic range, allowing the detection of miRNAs with plasma sample volumes as small as ~2.5 µL. Large dilutions are beneficial to significantly reduce concentrations of interfering substances in biomedical samples such as blood plasma and serum, and thus minimize cross-reactivity (Table 2), resulting in much lower background signals. The accuracy of the assay was validated through spike-recovery rates (Table 1) and low cross reactivity (Table 2), and confirmed by excellent correlations of the found concentrations of each miRNA from the FL assay with an ECL referee method (Supplementary Materials, Figure S2). Additionally, results on a small cohort of patient samples illustrate the capability to differentiate between Alzheimer-positive and Alzheimer-negative patients based on levels of analyzed miRNA biomarker from the assay.

In summary, we report above a new approach to develop an accurate, ultrasensitive, multiplexed, mix-and-read 3D-printed CRISPR well-plate assay using the first example of immobilized crRNA/CAS protein complexes to detect three ALZ-specific miRNAs in diluted plasma samples without amplification. This new 96-well-plate assay can measure standards and multiple samples in the same plate. Future goals include incorporating additional miRNA biomarkers related to ALZ and exploring novel multiple miRNA panels for cancer and cancer metastasis biomarkers, as well as developing modifications designed to measure both protein and miRNA biomarkers in the same CRISPR assay.

## Figures and Tables

**Table 1:** Spike recovery data for miRNAs from well-plate assay in 0.1% pooled human plasma (n = 3).

Spiked Conc, (fg/mL)	miRNA 30e-5p		miRNA 200c-3p		miRNA 34c-5p	
	Found (fg/mL)	Recovery %	Found (fg/mL)	Recovery %	Found (fg/mL)	Recovery %
70	83	118 ± 12	60	86 ± 8	79	112 ± 11
850	808	95 ± 9	902	106 ± 10	703	83 ± 7
3500	4224	120 ± 14	4100	117 ± 13	3380	96 ± 7
35,000	40,254	115 ± 11	40,790	116 ± 12	39,900	114 ± 7

**Table 2:** Cross-reactivity between different miRNAs in pooled human plasma miRNA.

miRNA	% Cross-Reactivity with CRISPR RNA		
	30e-5p	34c-5p	200c-3p
30e-5p		3.3	3.3
34c-5p	4.1		4.7
200c-3p	2.5	3.6	

Grey means no possibility of cross reactivity.

**Table 3:** Sample results.

Patient	34c-5p (ng/mL)	30e-5p (ng/mL)	200c-3p (ng/mL)	ALZ Diagnosis
1	0.000028	0.000023	0.000030	Negative
2	0.000041	0.000033	0.000044	Negative
3	0.655	0.0001834	0.965	Positive
4	2.16	3.11	1.02	Positive
5	2.74	6.52	1.21	Positive
6	3.74	21.2	1.69	Positive
7	9.12	86.4	3.11	Positive
8	24.2	349	3.01	Positive

## Data Availability

All relevant data are presented in this paper or Supplementary Materials.

## Supplementary Materials

The following supporting information can be downloaded at: https://www.mdpi.com/article/10.3390/bios15060346/s1, Figure S1: Well size optimization; Figure S2: Correlation plots with alternative method; Table S1: crRNA sequences. The document provides details on miRNA sequences and assay optimization related to Alzheimer’s disease (AD). List of target miRNAs (34c-5p, 30e-5p, 200c-3p, and a control) along with their associated crRNA sequences, except for the control, which lacks a crRNA. The development of a miRNA fluorescence assay focused on optimizing CRISPR RNA and Cas13a. For CRISPR well-plate optimization, the assay was tested using miRNA34c-5p across four well volumes. This finding supports the use of 50-μL wells as a cost-effective and resource-efficient option, reducing the required sample and reagent volumes without compromising performance. Reference [53] is cited in Supplementary Materials.
