# Supplementary material for: Multiplexed CRISPR Assay for Amplification-Free Detection of miRNAs
Source: Biosensors (Basel). 2025 May 29;15(6):346. doi: 10.3390/bios15060346 (PMC12190982; doi:10.3390/bios15060346)
Supplement: Supplementary file 1 [file biosensors-15-00346-s001.zip › biosensors-3590487-supplementary.pdf]

Multiplexed CRISPR Assay for Amplification-free Detection of miRNAs

P. I. Thilini De Silva[1, †], Keshani Hiniduma[1, †], R. Canete[1], Ketki S. Bhalerao[1], Sherif M. Shawky[1], Hansana Gunathilaka,[1] Jessica L. Rouge[1], Islam Mosa,[1] David C. Steffens[4], Kevin Manning[4], Breno S. Diniz[4,5] and James F. Rusling[1,2, 3, 6] *

[1] Department of Chemistry, University of Connecticut, Storrs, CT 06269, USA
[2] Institute of Material Science, University of Connecticut, Storrs, CT 06269, USA
[3] School of Chemistry, National University of Ireland at Galway, Galway, Ireland. H91 TK33
[4] Department of Psychiatry, UConn Health, Farmington, CT 06030
[5] UConn Center on Aging, UConn Health, Farmington, CT 06030
[6] Department of Surgery and Neag Cancer Center, UConn Health, Farmington, CT 06030
** Corresponding author: james.rusling@uconn.edu*
† These authors contributed equally to this work.

Supporting Information: Table of Contents

miRNA Sequences used in this work: 1

CRISPR well plate optimization 2

Correlation plots: Plots below were generated for the 8 patient samples using the FL assay vs. result for the same samples using a recently developed ECL assay. 3

References 3

miRNA Sequences used in this work:

Table S1: Target miRNA and Associated crRNA sequence associated with Alzheimer's disease (AD)[1,2,3]

Name	Target miRNA sequence	Associated crRNA
34c-5p	5' rArGrG rCrArG rUrGrU rArGrU rUrArG rCrUrG rArUrU rGrC 3'	5' rArCrT rArCrC rCrCrA rArArA rArCrG rArArG rGrGrG rArCrT rArArA rArCrG rCrArA rUrCrA rGrCrU rArArC rUrArC rArCrU rGrCrC rU 3'
30e-5p	5' rUrGrU rArArA rCrArU rCrCrU rUrGrA rCrUrG rGrArA rG 3'	5' rArCrT rArCrC rCrCrA rArArA rArCrG rArArG rGrGrG rArCrT rArArA rArCrC rUrUrC rCrArG rUrCrA rArGrG rArUrG rUrUrU rArCrA 3'
200c-3p	5' rUrArA rUrArC rUrGrC rCrGrG rGrUrA rArUrG rArUrG rGrA 3'	5' rArCrT rArCrC rCrCrA rArArA rArCrG rArArG rGrGrG rArCrT rArArA rArCrU rCrCrA rUrCrA rUrUrA rCrCrC rGrGrC rArGrU rArUrU rA 3'
Control	5' rGrUrC rGrCrU rArCrA rGrArC rArCrU rGrGrC rUrGrC rArG 3'	N/A

CRISPR well plate optimization

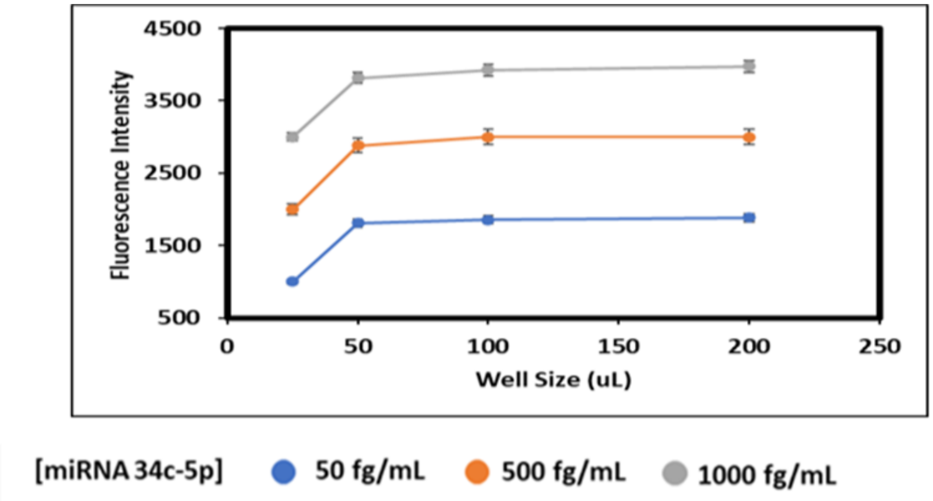

Figure S1. Well size optimization showing results from miRNA 34c-5p detection at three different concentrations (50, 500, and 1000 fg/mL). Fluorescence intensity was measured across varying well volumes (25, 50, 100, and 200 µL) to determine the optimal assay conditions.

Well size was optimized by conducting the assay across different concentrations of the analyte miRNA (miRNA30e-5p), spanning the desired dynamic range. The assay was tested using four different well volumes: 200 µL (original size of a 96-well plate), 100 µL, 50 µL, and 25 µL. The resulting plots indicate that signal saturation occurs at a volume of 50 µL. The fluorescence signal intensity between the 200 µL and 50 µL plates shows only a 4% per mL average difference, demonstrating that the 50 µL volume is suitable for the assay. This reduced volume effectively decreases the amount of sample and reagents required, making it a more cost-effective and resource-efficient option.

Correlation plots: Plots below were generated for the 8 patient samples using the FL assay vs. result for the same samples using a recently developed ECL assay.[4]

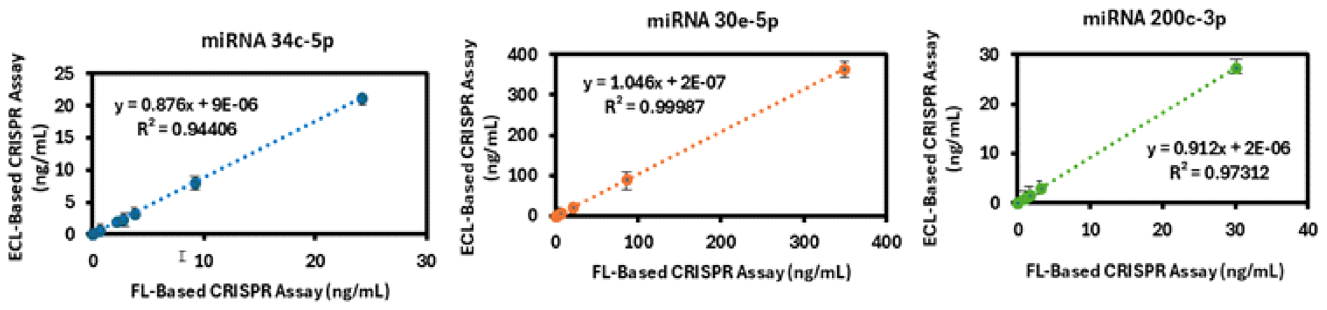

Figure S2. Correlation plots FL assay and a recently developed ECL assay for 3 different miRNAs for patient sample analysis (a) miRNA 34c-5p (b) miRNA 30e-5p (c) miRNA 200c-3p in. (quantified in ng/mL, n = 3) (ref. 4)

References

1. Cogswell, J. P.; Ward, J.; Taylor, I. A.; Waters, M.; Shi, Y.; Cannon, B.; Kelnar, K.; Kemppainen, J.; Brown, D.; Chen, C.; Prinjha, R. K.; Richardson, J. C.; Saunders, A. M.; Roses, A. D.; Richards, C. A. Identification of miRNA Changes in Alzheimer's Disease Brain and CSF Yields Putative Biomarkers and Insights into Disease Pathways. Journal of Alzheimer's Disease 2008, 14 (1), 27–41. https://doi.org/10.3233/jad-2008-14103

2. Müller, M.; Kuiperij, H. B.; Claassen, J. A.; Küsters, B.; Verbeek, M. M. MicroRNAs in Alzheimer's disease: differential expression in hippocampus and cell-free cerebrospinal fluid. Neurobiology of Aging 2014, 35 (1), 152–158. https://doi.org/10.1016/j.neurobiolaging.2013.07.005.

3. Swarbrick, S.; Wragg, N.; Ghosh, S.; Stolzing, A. Systematic review of MIRNA as biomarkers in Alzheimer's Disease. Molecular Neurobiology 2019, 56 (9), 6156–6167. https://doi.org/10.1007/s12035-019-1500-y.

4. Hiniduma, K.; De Silva, P. I. T.; Canete, R.; Gunathillaka, H.; Clement, O.; Vora, P.; Shawky, S. M.; Rouge, J. L.; Mosa, I.; Breno, D.; Rusling, J. F. ECL-CRISPR Array for Multiplexed Detection of miRNAs. ACS Sensors, Ms. submitted.
